# Mechanism of effective orthotic therapy for the painful cavus foot

**DOI:** 10.1186/1757-1146-6-S1-O3

**Published:** 2013-05-31

**Authors:** Bijan Najafi, James Wrobel, Joshua Burns

**Affiliations:** 1 Interdisciplinary Consortium on Advanced Motion Performance (iCAMP), Southern Arizona Limb Salvage Alliance (SALSA)/Arizona Center on Aging, University of Arizona College of Medicine, Tucson, AZ, USA; 2 Internal Medicine; Metabolism, Endocrinology and Diabetes Division, University of Michigan Medical School, Ann Arbor, MI, USA; 3 The University of Sydney and The Children’s Hospital at Westmead, Sydney, Australia

## Background

People who have extremely high-arched feet or pes cavus often suffer from substantial foot pain. Custom-made foot orthoses have been shown to be an effective treatment option, but their specificity is unclear. It is generally thought that one of the primary functions of custom foot orthoses is redistribution of abnormal plantar pressures. This study sought to identify variables associated with pain relief after custom foot orthoses intervention.

## Methods

Demographic, physical characteristics and Pedar^®^ in-shoe plantar pressure data from a randomised controlled trial of 154 participants with painful pes cavus were retrospectively re-analysed at baseline and three month post orthoses intervention. The participants were randomised to a treatment group prescribed custom-made foot orthoses or a control group given sham orthoses.

## Results

No relationship between change in pressure magnitude and change in symptoms was found in either group. While redistribution of plantar pressure, measured with the Dynamic Plantar Loading Index, had a significant effect on pain relief (*p*=0.03). Our final model predicted 73% of the variance in pain relief from custom foot orthoses and consisted of initial pain level, BMI, foot alignment, and changes in both Dynamic Plantar Loading Index and pressure-time integral. Results indicate that a primary function of effective orthotic therapy is redistribution of abnormal plantar pressures.

## Conclusion

This study provides the mechanism by which custom-made foot orthoses reduce pain and disability in patients with painful pes cavus. The proposed model may assist in better designing and assessing orthotic therapy for pain relief in patients with a variety of painful foot disorders.

